# Estrone sulphate uptake by the microvillous membrane of placental syncytiotrophoblast is coupled to glutamate efflux

**DOI:** 10.1016/j.bbrc.2018.10.074

**Published:** 2018-11-17

**Authors:** Emma M. Lofthouse, Jane K. Cleal, Ita M. O'Kelly, Bram G. Sengers, Rohan M. Lewis

**Affiliations:** a University of Southampton, Faculty of Medicine, UK; b University of Southampton, Faculty of Engineering and the Environment, UK; c University of Southampton, Institute for Life Science, UK

**Keywords:** Organic anion transporting polypeptides, Counter-ions, Glutamate, Thyroid hormone, Bile acids

## Abstract

Organic anion transporters (OATs) and organic anion transporting polypeptides (OATPs) are transport proteins that mediate exchange of metabolites, hormones and waste products. Directional transport by these transporters can occur when exchange is coupled to the gradients of other substrates. This study investigates whether the activity of OATP4A1 and OATP2A1 on the maternal facing microvillus membrane of the placental syncytiotrophoblast is coupled to the glutamate gradient. OAT and OATP transporter proteins were over expressed in *Xenopus* oocytes to study their transport characteristics. Further transport studies were performed in term human placental villous fragments. *Xenopus* oocytes expressing OATP4A1 mediated glutamate uptake. No glutamate transport was observed in oocytes expressing OAT1, OAT3, OAT7 or OATP2A1. In oocytes expressing OATP4A1, uptake of estrone sulphate, thyroid hormones T3 and T4 and the bile acid taurocholate stimulated glutamate efflux. In term placental villous fragments addition of estrone sulphate and taurocholate trans-stimulated glutamate efflux. Coupling of OATP4A1 to the glutamate gradient may drive placental uptake of estrone-sulphate and thyroid hormone while also facilitating uptake of potentially harmful bile acids. In contrast, if OATP2A1 is not coupled to a similar gradient, it may function more effectively as an efflux transporter, potentially mediating efflux of prostaglandins to the mother. This study provides further evidence for glutamate as an important counter-ion driving transport into the placenta.

## Highlights

• OATP4A1 and OATP2A1 are present on the maternal facing surface of the placenta.
• OATP4A1, but not OATP2A1, activity was coupled to glutamate efflux.
• Placental estrone-sulphate and thyroid hormone uptake is coupled to glutamate efflux.
• Glutamate acts as a counter-ion for OATP4A1, driving transport into the placenta.

## 1 Introduction

The placenta is a multifunctional organ, which acts as a selective barrier between the mother and placenta. Placental transport of nutrients, metabolites, waste products and hormones is essential for fetal survival. Impaired placental function, in terms of impaired nutrient delivery or removal of waste products, may impair fetal development.

The organic anion transporters (OATs) and organic anion transporting polypeptides (OATPs) are two membrane protein families whose members transport a diverse range of endogenous molecules and xenobiotics, including therapeutic agents, into and out of cells [1]. In the placenta, OATs and OATPs mediate transport of metabolites such as steroid sulphates, thyroid hormones and prostaglandins as well as transport of waste products and xenobiotics. In the human placenta OATP4A1 (SLC04A1) and OATP2A1 (SLC02A1) are expressed on the maternal facing microvillous membrane and OAT4 and OATP2B1 on the fetal facing basal membrane of the placental syncytiotrophoblast [2,3].

OATs and OATPs are typically exchange transport proteins that transport a substrate together with the reverse transport of a counter-ion. The ability of these transporters to accumulate a substrate within a cell (or conversely mediate net efflux) is dependent on the gradient of the counter-ion or ions, across the membrane [4,10]. While exchangers are considered to be obligate exchangers we have demonstrated a low level of non-obligate transport in the amino acid transporter LAT2 [5]. We have previously shown that placental uptake of substrates from the fetus by OAT4 and OATP2B1 on the basal membrane is coupled to glutamate efflux down its gradient [6]. As placental intracellular glutamate concentrations are high, 5–10 mM [7,8], glutamate will provide a constant driver for uptake of waste products from the fetus [6].

On the maternal facing microvillous membrane, OATP2A1 and OATP4A1 are reported to be expressed but neither has been shown to transport glutamate and it is not clear what gradients drive uptake (or efflux) by these transporters [9,10]. OATP2A1 predominantly transports prostaglandins as well as steroid hormone substrates like dehydroepiandrosterone (DHEAS) [11]. Placental oestrogen synthesis is important to maintain the pregnancy [12]. OATP4A1 predominantly transports the bile acid taurocholate, the thyroid hormone triiodothyronine (T3) prostaglandins and estrone sulphate [1]. The role of these transporters on the microvillous membrane may be to transport metabolites for steroid synthesis for the placenta.

Understanding the gradients driving secondary active transport by OATs and OATPs on the microvillous membrane would provide insight into their function in human placenta. This study explores the role of glutamate as a counter-ion for the OATPs expressed on the maternal facing apical membrane of the placental syncytiotrophoblast.

## 2 Methods

### 2.1 cRNA synthesis for microinjection into oocytes

Plasmids containing the cDNA of human SLC22A6 (OAT1), SLC22A8 (OAT3), SLC22A9 (OAT7), SLC02A1 (OATP2A1), SLC02B1 (OATP2B1), and SLC04A1 (OATP4A1) obtained from Origene (Origene, USA) were linearized using restriction enzymes (Promega, Southampton, UK). Plasmids containing two T7 promoter sites were double digested before purification to prevent synthesis of cRNA from the non-coding T7 site. The OATP4A1 plasmid was digested with the restriction enzymes NdeI and StuI. The OATP2B1 plasmid was digested with the restriction enzymes NdeI and XhoI. cRNA was synthesized from the coding T7 promoter using the Ambion mMachine mMessage kit according to the manufactures instructions (Life Technologies, UK).

### 2.2 Microinjection into *Xenopus oocytes*

*Xenopus laevis* oocytes were obtained from the European *Xenopus* Resource Centre (Portsmouth, UK). Oocytes were treated with collagenase (2 mg/ml) in buffer OR2 (2.5 mM KCl, 82.5 mM NaCl, 1 mM CaCl_2_, 1 mM Na_2_HPO_4_, 1 mM MgCl_2_, 5 mM HEPES) for 1 h at room temperature. Oocytes were then incubated in ND91 buffer (2 mM KCl, 91 mM NaCl, 1.8 mM CaCl_2_, 1 mM MgCl_2_, 5 mM HEPES, 1% Pen/Strep and 0.1% Gentamycin Sulphate) overnight at 18 °C. Stage V oocytes were injected with 20 μg of the cRNA for the transporter of interest dissolved in 56 nl of water. The water-injected control oocytes were injected with an equivalent volume of water. Studies were performed two to three days after injection of the relevant transporter cRNA.

### 2.3 OAT and OATP mediated uptake of glutamate into Xenopus oocytes

Initial OATP4A1 uptake time-course experiments were performed to show the ^3^H-estrone sulphate (^3^H-ES) and ^14^C-glutamate uptake at 5, 10 and 20 min and uptake experiments were conducted at 10 min. Uptake experiments were conducted with 75 nM ^14^C-glutamate or 11 μM ^3^H-ES alone or in the presence of 2.5 mM glutamate, estrone sulphate (ES), bromosulphothalein (BSP) or alpha ketoglutarate (10 replicate oocytes, n = 3 ovaries).

Uptake of ^14^C-tracer into the media was measured by liquid scintillation counting (Packard-Perkin Elmer, Massachusetts USA).

### 2.4 *OAT and OATP mediated efflux of glutamate from* Xenopus *oocytes*

Oocytes were preloaded with ^14^C-glutamate by incubating for 30 min in 20 μM (50 μCi/l) ^14^C-glutamate in ND91 buffer and then washed 3 times in 1 ml of ND91 buffer to remove extracellular label. Initial time-course experiments were performed to show the ^14^C-glutamate efflux at 2, 5 and 10 min (5 oocytes per condition, triplicate conditions, n = 3 ovaries). For OATP4A1 time-courses, oocytes where trans-stimulated by addition of 2.5 mM glutamate to the extracellular ND91 buffer.

Based on time-course experiments, further experiments were performed at 10 min. Efflux of ^14^C-tracer into the media was measured by liquid scintillation counting (Packard-Perkin Elmer, Massachusetts USA). Each independent experiment used oocytes from a different frog and were conducted in different weeks. Within each experiment, all conditions were performed with multiple oocytes and in triplicate.

To determine whether OATP4A1 mediates glutamate efflux, OATP4A1 and water injected oocytes were injected with 200 μmol ^14^C-glutamate on the day of the efflux experiment. Each oocyte was then washed twice in sodium free ND91 before being incubated with sodium free ND91 alone, 2.5 mM glutamate, 2.5 mM estrone sulphate, 2.5 mM BSP, 100 μM thyroxine (T4), 100 μM triiodothyronine (T3) or 2.5 mM glycine to stimulate efflux of ^14^C-glutamate (5 oocytes per substrate in triplicate, n = 5 ovaries). Efflux was stopped after 5 min and the efflux buffer of 5 oocytes was combined and analysed by liquid scintillation counting.

### 2.5 Efflux of ^14^C glutamate from human term placenta villous fragments

Human placentas were collected from daytime full-term caesarean deliveries from uncomplicated pregnancies at the Princess Anne Hospital in Southampton, in accordance with ethical approval from the Southampton and Southwest Hampshire Regional Ethics Committee (approval no. 11/sc/0323).

Placentas were collected as soon as possible after delivery and fragments (≈10 mg) dissected from the villous tissue and placed in Tyrode's buffer (duplicate conditions, 3 fragments per replicate, n = 3 placentas). Fragments were preloaded with ^14^C-glutamate by incubating for 60 min in 20 μM (50 μCi/l) ^14^C-glutamate in Tyrode's buffer at 37**°**C and then washed 3 times in 1 ml of Tyrode's buffer to remove extracellular label. Preloaded fragments were then incubated with buffer alone of buffer containing estrone sulphate or taurocholate for 5 min. The fragments were removed and the ^14^C-glutamate efflux into the buffer determined by liquid scintillation counting (Packard-Perkin Elmer, Massachusetts USA).

### 2.6 Statistics

^3^H-estrone sulphate uptake in oocytes and placental villous fragments was analysed by a one way ANOVA with a Dunnett's post hoc test in which ^3^H-estrone sulphate uptake in the presence of OAT substrates was compared to ^3^H-estrone sulphate alone.

^14^C-glutamate efflux in OATP4A1 oocytes and villous fragments were analysed by a one way ANOVA with a Dunnett's post hoc test in which ^14^C-glutamate efflux stimulated by OATP4A1 substrates was compared to ^14^C-glutamate efflux in response to buffer alone. Significance was assumed at P < 0.05 and oocyte data are adjusted for water injected responses and presented as mean ± SEM.

^3^H-estrone sulphate uptake in OATP2A1 injected oocytes were analysed by two-way ANOVA and presented as mean and SEM and adjusted for water injected responses.

## 3 Results

### 3.1 Glutamate uptake in oocytes

**Fig. 1:**
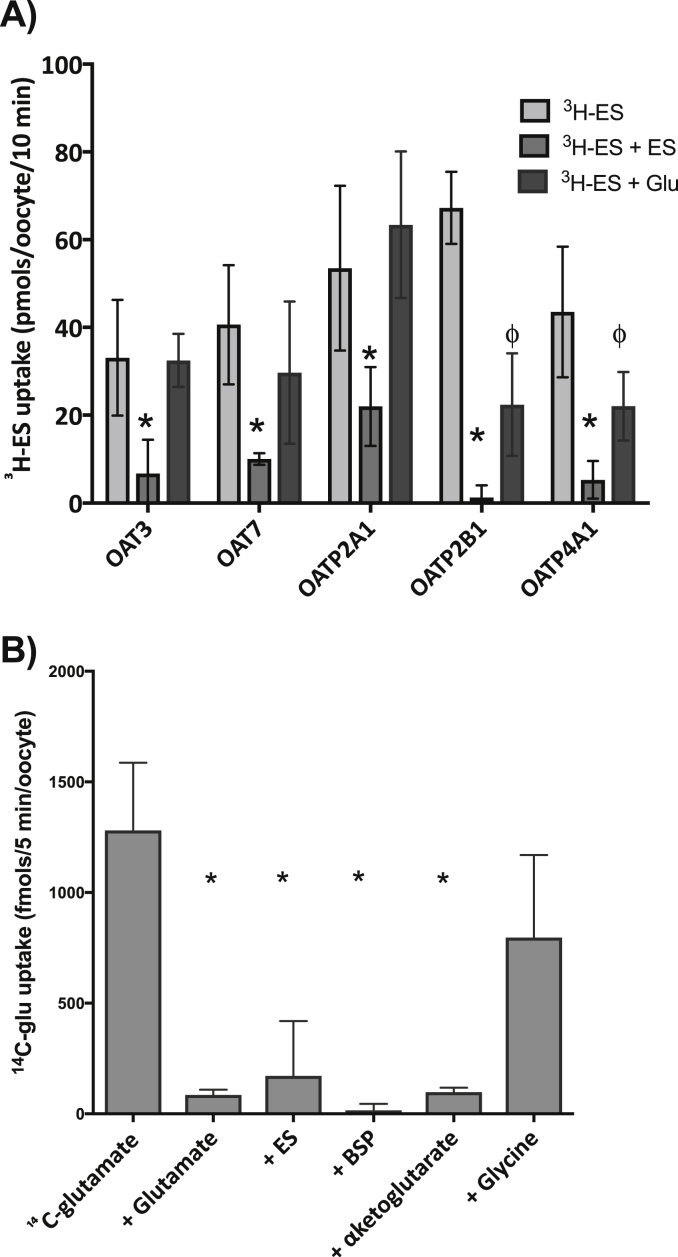
OATP4A1 mediates glutamate uptake **A**) OATP2B1 and OATP4A1 are inhibited by glutamate. ^3^H-ES uptake in transporter expressing oocytes was inhibited by 2.5 mM ES (*p < 0.05, n = 3 experiments, 10 oocytes per condition). 2.5 mM glutamate (Glu) inhibited ^3^H-ES uptake in OATP2B1 and OATP4A1 expressing oocytes (ϕ p < 0.001). Data are adjusted for water injected oocyte responses. **B**) OATP4A1 mediated ^14^C-glutamate uptake is inhibited by OATP4A1 substrates. Under sodium free conditions, ^14^C-glutamate uptake is inhibited by 2.5 mM glutamate estrone sulphate, BSP and alpha ketoglutarate (*p < 0.05, n = 3 ovaries). Data are adjusted for water injected background responses and presented as mean (SEM).

In *Xenopus* oocytes expressing OAT3, OAT7, OATP2A1, OATP2B1, and OATP4A1, ^3^H-ES uptake was inhibited by ES indicating that the transporters were expressed in the oocyte and were functional (p < 0.05, n = 3 individual ovaries, 10 oocytes per condition) (Fig. 1a). In oocytes expressing OATP4A1 and OATP2B1, ^3^H-ES uptake was inhibited by glutamate (Fig. 1a). In oocytes expressing OATP4A1, uptake of ^14^C-glutamate (under sodium free conditions to block transport by excitatory amino acid transporters) was inhibited by 2.5 mM glutamate, ES, BSP and alpha-ketoglutarate but not by the negative control glycine (p < 0.05, n = 3 individual ovaries, 10 oocytes per condition) (Fig. 1b).

### 3.2 Glutamate efflux in oocytes

**Fig. 2:**
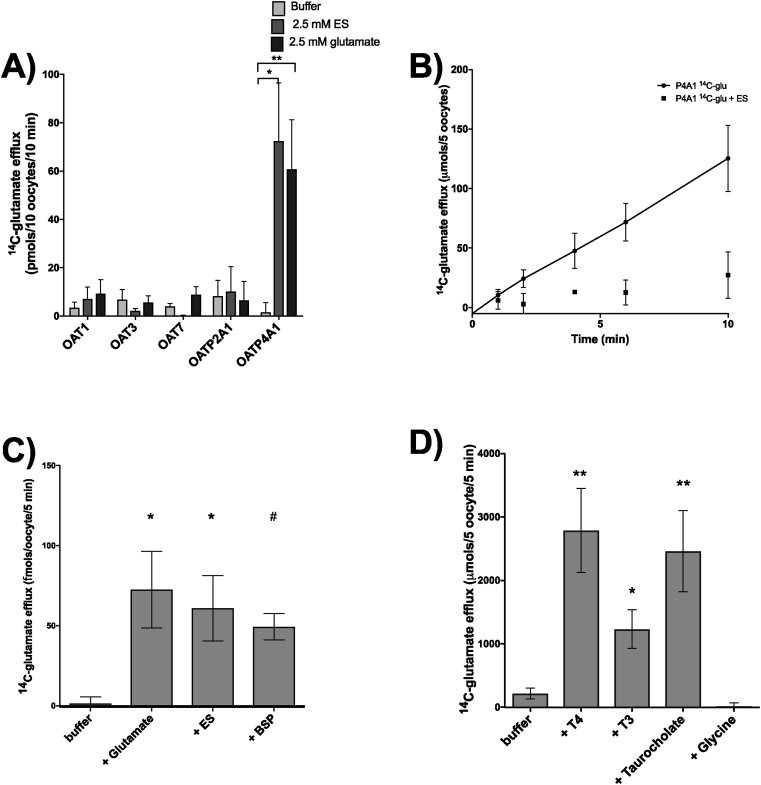
Glutamate efflux is coupled to uptake of thyroid hormone and taurocholate in OATP4A1 expressing *Xenopus* oocytes. **A**) ^14^C-glutamate efflux was stimulated by 2.5 mM estrone sulphate, (*p = 0.03) and 2.5 mM glutamate (**p < 0.01) in OATP4A1 expressing oocytes but not by OAT1, OAT3, OAT5, OAT7 or OATP2A1 (n = 3 individual ovaries, 10 oocytes per condition). **B**) ^14^C-glutamate efflux time course from 0 to 10 min in OATP4A1 expressing oocytes. ^14^C-glutamate efflux is significantly higher compared to water injected control oocytes (p = 0.05, n = 3 individual ovaries, 5 × 3 oocytes per condition). **C**) ^14^C-gluatamte efflux was stimulated by 2.5 mM glutamate (*p = 0.03) and 2.5 mM estrone sulphate (*p = 0.01) although stimulation by 2.5 mM BSP did not reach statistical significance (#p = 0.08) under sodium free conditions (n = 5 individual ovaries, 5 × 3 oocytes per condition). **D**) ^14^C-gluatamte efflux is stimulated by OATP4A1 substrates 100 μM thyroxine (T4, **p < 0.001), 100 μM triiodothyronine (T3, *p = 0.014) and 2.5 mM taurocholate (**p < 0.001) but not by glycine (n = 5 individual ovaries, 5 × 3 oocytes per condition). Data are adjusted for background water injected responses and presented as mean and SEM.

In *Xenopus* oocytes expressing OAT1, OAT3, OAT7 or OATP2A1 and preloaded with ^14^C-glutamate there was no efflux of glutamate following trans-stimulation with ES or glutamate (Fig. 2a). In *Xenopus* oocytes expressing OATP4A1 and preloaded with ^14^C-glutamate, both glutamate and ES trans-stimulated ^14^C-glutamate efflux (p < 0.05, n = 3 ovaries, 10 oocytes per condition) (Fig. 2a). Glutamate efflux from OATP4A1 expressing oocytes increased in a linear manner with time (Fig. 2b). In OATP4A1 expressing *Xenopus* oocytes, the known OATP4A1 substrate ES (p = 0.012, n = 3 ovaries, 3 × 5 oocytes per condition) and glutamate (p = 0.03, n = 3 ovaries, 3 × 5 oocytes per condition) trans-stimulated efflux of ^14^C-glutamate under sodium free conditions with a trend towards stimulation of efflux by BSP (p = 0.08 n = 3 ovaries, 3 × 5 oocytes per condition, Fig. 2c). Compared to buffer alone, OATP4A1 substrates thyroxine (T4, p < 0.001), T3 p = 0.014, n = 3 ovaries, 3 × 5 oocytes per condition) and taurocholate (p < 0.001, n = 3 ovaries, 3 × 5 oocytes per condition) also trans-stimulated ^14^C-glutamate efflux, but non-substrate glycine did not (Fig. 2d).

### 3.3 Placental villous fragment experiments

**Fig. 3:**
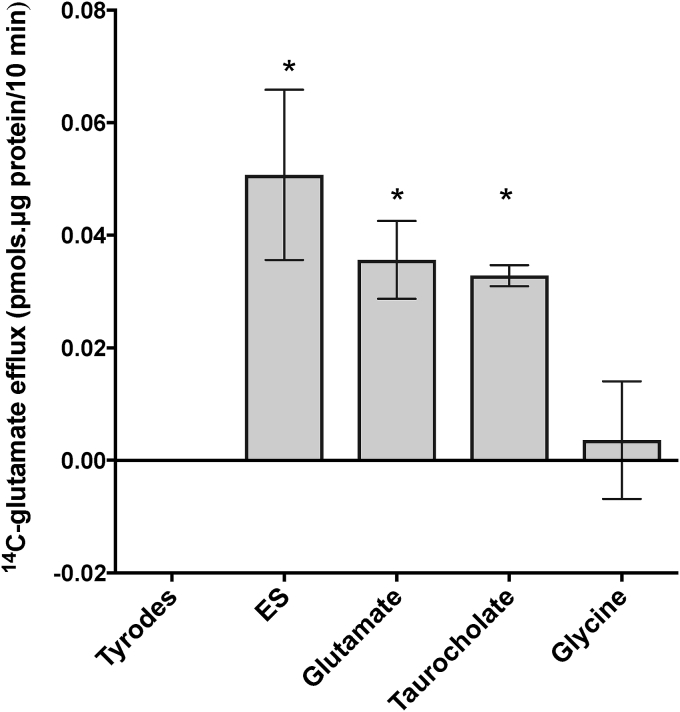
Uptake of OATP4A1 substrates is coupled glutamate efflux in placental villous fragments. 1 mM OATP4A1 substrates; estrone sulphate, glutamate and taurocholate but not by glycine (p < 0.05, n = 5 experiments, 3 fragments per condition, 3 replicates). Data are presented as mean and SEM.

In human placental villous fragments preloaded with ^14^C-glutamate, extracellular glutamate and the known OATP4A1 substrates ES and taurocholate (1 mM) trans-stimulated efflux of ^14^C glutamate (p < 0.05, n = 5 experiments, 3 fragments per condition, 3 replicates) (Fig. 3).

### 3.4 Trans-stimulation experiments in oocytes

**Fig. 4:**
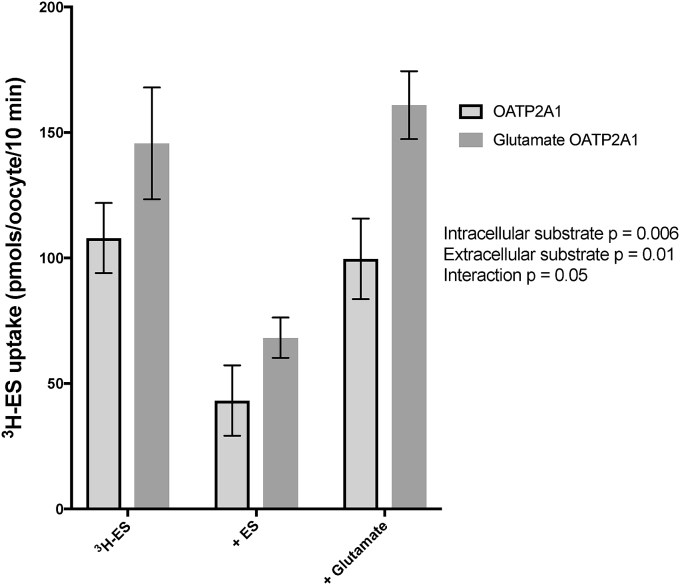
Intra-oocyte glutamate injection trans-stimulates estrone sulphate uptake in oocytes expressing OATP2A1 despite the fact that glutamate does not appear to be an OATP2A1 substrate. Compared to ^3^H-ES uptake alone in OATP2A1 oocytes, uptake was inhibited by cold ES, but not by glutamate. The injection of 5 mM glutamate into OATP2A1 expressing oocytes resulted in a trans-stimulation of ^3^H-ES uptake (p = 0.006). Data are analysed by two-way ANOVA and presented as mean and SEM and adjusted for water injected responses (n = 3 individual ovaries, 5 oocytes per condition).

^3^H-ES uptake into *Xenopus* oocytes expressing OATP2A1 that had been pre-injected with water or a final concentration of 5 mM glutamate was inhibited by extracellular 1 mM ES but not by 1 mM glutamate (p = 0.01) ^3^H-ES uptake was trans-stimualted by intracellular glutamate injection (p = 0.006). There was no interaction between oocyte injection and extracellular conditions (p = 0.5) (Fig. 4).

## 4 Discussion

This study demonstrates that on the microvillous membrane of the placental syncytiotrophoblast OATP4A1, but not OATP2A1, can use glutamate as a counter-ion. This has implications for the directionality of transport by these transporters, as high intracellular glutamate concentrations will energetically favour uptake by OATP4A1 compared to OATP2A1. This will facilitate uptake of important substrates such as thyroid hormone but could also have detrimental effects, for instance driving uptake of bile acids in intrahepatic cholestasis of pregnancy.

**Fig. 5:**
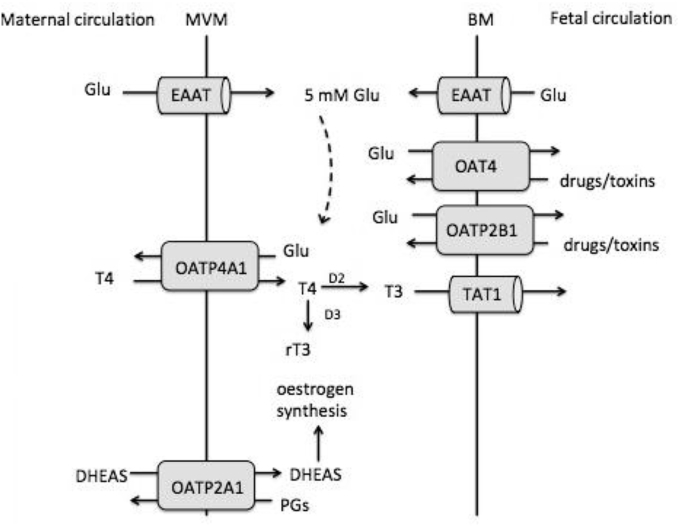
OATs/OATPs in the syncytiotrophoblast and their interaction with substrates and other transport systems. Uptake of thyroid hormone by OATP4A1 would allow its transport to the fetus by TAT1 (*SLC16A10*). If OATP2A1 is not coupled to an outwardly directed gradient it would be able to act as an efflux transporter. Uptake of DHEAS by OATP2A1 will facilitate placental oestrogen synthesis.

OATP4A1 was shown to exchange known substrates for glutamate. This indicates that in the placenta the glutamate gradient will drive the uptake of prostaglandins, oestrogen precursors, thyroid hormones and bile acids. Uptake of thyroid hormone into the placenta by OATP4A1 may be important for placental and fetal metabolism (Fig. 5) [13]. Thyroid hormone taken up by OATP4A1 on the microvillous membrane could then diffuse to the fetus via the transporter TAT1 that is localised to the basal membrane of placental syncytiotrophoblast [14]. In contrast, OATP4A1 could mediate pathophysiological transport of bile acids in intrahepatic cholestasis in pregnancy [15].

This study demonstrates that glutamate is not a counter-ion for OATP2A1 making it the only known OAT or OATP in the placenta not coupled to glutamate. Assuming it is not coupled to another outwardly directed gradient, OATP2A1 could more easily mediate efflux of substrates from the placenta to the mother. The placenta synthesizes and secretes prostaglandins and OATP4A1 could mediate release of prostaglandins to the maternal circulation in exchange for other substrates that the placenta needs from the maternal circulation such as DHEAS [12,16]. While it is unlikely that OATP2A1 would primarily exchange prostaglandins for DHEAS in the placenta, it is an example of how co-operative arrangements could occur to provide counter-ions necessary for exchanger function.

The apparent trans-stimulation of estrone sulphate uptake by OATP2A1 following intra-oocyte glutamate injection is interesting, given that glutamate does not appear to be an OATP2A1 substrate. The most likely explanation is that a metabolite of glutamate is a counter-ion for OATP2A1 activity although further work is required to demonstrate this. The transporter OAT1 has previously been reported to transport glutamate derivatives [17].

The availability of counter-ions for these transporters may regulate their activity. For OATP4A1 high intracellular glutamate level would suggest it always operates at maximal activity, as intracellular glutamate would rapidly recycle from the active face of the transporter to the extracellular face of the membrane. However, if the counter-ion of OATP2A1 is of a lower abundance metabolite then the activity of the transporter could be linked to the rate of metabolism as the availability of counter-ions may control the rate at which its binding site was recycled to the outward configuration.

Identifying the counter-ions for these transporters would help clarify their physiological roles and activity. The non-placental OATs and OATPs that were tested did not transport glutamate. While OATP4A1 is ubiquitously expressed the other OATs and OATPs that transport glutamate tend to be expressed in tissues involved in clearing substrates from the circulation such as the kidney, liver and placenta [18,19]. As the majority of cell types contain high concentrations of glutamate, for those OATs/OATPs that do transport glutamate this will favour the direction of drug transport into the cell. The primary counter-ions used by members of the OAT and OATP families remain poorly defined and answering this question may provide important insights into the physiology of these transporters.

It is significant that three of the four OATs and OATPs which mediate glutamate transport are expressed in the placenta and this suggests the glutamate gradient plays an important role in this cell type. Coupling transport to the glutamate gradient will favour uptake of extracellular substrates and on the basal membrane glutamate efflux will drive uptake of fetal substrates by both OAT4 and OATP2B1 [6]. On the microvillous membrane OATP4A1 is coupled to glutamate favouring uptake of maternal substrates while we propose that OATP2A1 may be acting as an efflux transporter.

## Conflicts of interest

The authors have declared that no conflict of interest exists.
